# Open Clavicle Fracture: A Case Report

**DOI:** 10.31729/jnma.v63i292.9253

**Published:** 2025-12-31

**Authors:** Anjan Dhoj, Sakar Raj Pandey, Abhishek Kumar Thakur

**Affiliations:** 1 Institute of Medicine, Mahrajgunj Medical Campus, Maharajgunj, Kathmandu, Nepal

**Keywords:** *clavicle*, *debridement*, *fractures*, *open*

## Abstract

Open clavicle fractures are rare, accounting for only 1.8% of all fractures. This report describes a 37 year old male with an open Gustilo Anderson grade II midshaft clavicle fracture (Robinson type 2B1) caused by a wood cutting machine. He was hemodynamically stable but had an 8*6 cm wound with exposed clavicle. Initial emergency care included tetanus prophylaxis, intravenous antibiotics, and wound debridement. Definitive fixation was performed on day four using an 8 hole anatomical locking plate with screws. Postoperatively, progressive physiotherapy led to uneventful wound healing and good functional recovery. The case emphasizes that open clavicle fractures should be managed like open long bone fractures, with timely debridement, adequate antibiotics, and early surgical fixation, but simple arm pouch sling immobilization is sufficient until definitive fixation. This report contributes to the limited literature on open clavicle fracture management and supports operative stabilization for favorable outcomes in similar injuries.

## INTRODUCTION

Clavicle fracture accounts for 2.6 to 5% of all orthopedic fracture.^1^ Clavicle is subcutaneous in its middle third.^1^ Despite its common occurrence and subcutaneous status incidence of open fracture is very low nearly 1.8%.^2^ Open fracture is usually observed in younger population following high energy trauma or following medial fracture fragment indenting and gradually perforating the skin.^2^ Dictum of management is same as that for other open fracture i.e. antibiotics, irrigation and debridement and finally fixation.^2^ Open clavicle fracture however cannot be treated like closed ones with early definitive fixation and wound care should be given priority before definitive fixation in order to avoid complications like wound site infection. Usually healing is uneventful after surgical management.^2,3^ Here we present a case of open fracture in a male of 37 years presented with open clavicle fracture after sustaining injury due to wood-cutting machine within 2 hours managed with index debridement followed by definitive internal fixation with anatomical plates and screws.

## CASE REPORT

A 37 year old man with no known comorbidities presented to the emergency department with an alleged history of accidental cut injury over the right clavicular region with a wood cutting machine. He complained of pain and open wound over the right midclavicular region following the incident. He was hemodynamically stable with intact neurovascular examination and normal respiratory function, there was no signs of respiratory distress and his respiratory rate was 22 breaths per minute with oxygen saturation of 98% in room air. Cut injury measuring approximately 8x6 cm over midclavicular region with exposed clavicular bone was present.

**Figure 1:**
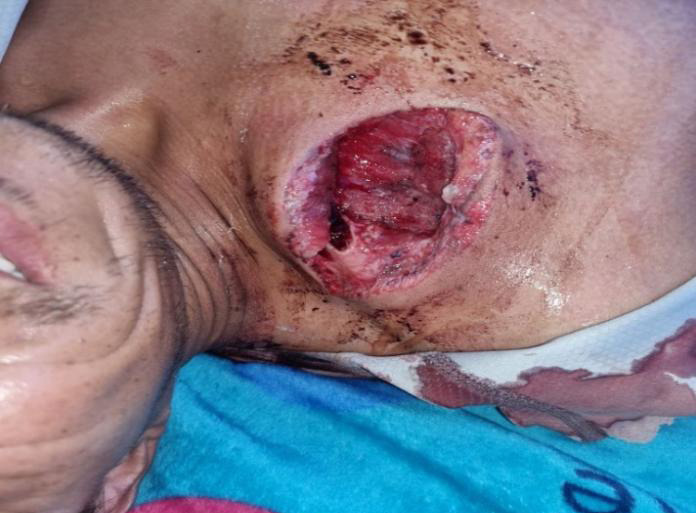
Pre-operative clinical image showing open clavicle fracture of Gustilo Anderson grade II.

Initially the wound was washed thoroughly and Tetanus toxoid was given at the emergency department. Following that chest and clavicle anteroposterior radiographs revealed a midshaft fracture of right clavicle. Then debridement was done and wound was left open with sterile dressing in OT under asceptic precautions and patient was admitted with prophylactic IV antibiotics (Cefuroxime 750mg TDS for 2 weeks, Metronidazole 500mg TDS for 2 weeks and Amikacin 500mg OD for 5 days). The fracture was classified as 2B1 according to Robinson’s.^4^ Total blood count and inflammatory markers were all within normal limit. As pus formation would generally take upto 3 day, definitive fixation was planned to be delayed atleast till the fourth day. The wound was inspected every day prior to OT and dressing was done, as the wound appeared healthy and there was no discharge from the wound site no culture samples were taken. The wound showed no signs of inflammation and healthy granulation tissues started appearing. Once we were satisfied with the wound status the patient was taken to the operating room in the fourth day and after giving general anesthesia was positioned in operating table in a beach chair position. We performed thorough debridement of the wound with normal saline and povidone iodine solution followed by medial and lateral extension of the wound centering over the clavicle and internal fixation with 8 hole anatomical locking plate with 3 medial and 4 lateral locking screws in respect to the fracture site. Postoperatively the shoulder was immobilized in a sling followed by progressive active-assisted physiotherapy as per standard in any other clavicle fracture. Wound dressing done on 3rd postoperative day revealed healthy wound, frequent wound inspections and dressing was done every third day as we were concerned about the potential infection at the wound site. Sutures were removed on the 14^th^ post-operative day and there was no wound related complications including signs of inflammation and discharge from the wound site till suture removal. Antibiotics were discontinued after two weeks and besides the use of triple antibiotics no other post operative treatment protocol was different from other clavicle fractures. As open fractures are notorious for infections usually in the first few days to weeks following surgery we were initially focused on preventing the infection and till the sixth weeks we followed up the patient there were no clinical signs and inflammatory markers suggestive of infection over the wound site.

**Figure 2:**
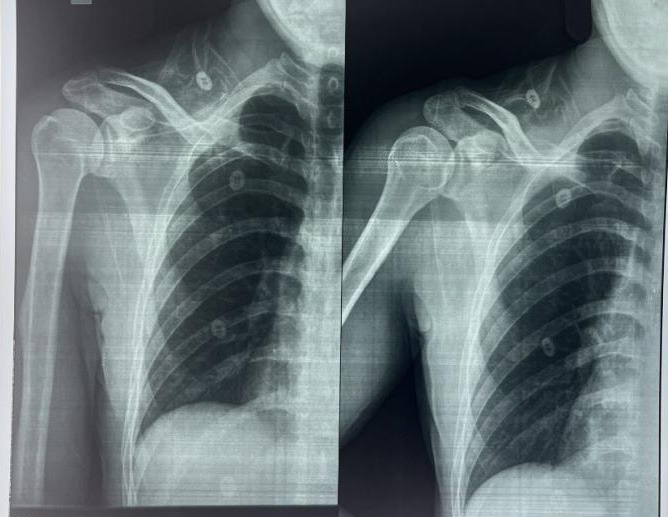
Pre-operative Xray.

**Figure 3:**
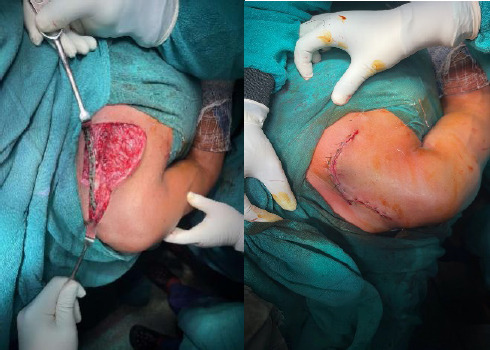
Intraoperative after plate placement and closure.

## DISCUSSION

Clavicle fracture occurrence is 2.6% to 5% of all orthopedics fractures which we come across.^1^ It accounts for two fifth of the shoulder girdle injuries. Anatomically shaft of clavicle occupying three fifth of middle portion is subcutaneous with it being covered only by myofascial layer comprising of platysma.^1^ Despite that observing open clavicle fracture is rare with 1.8% of all fracture.^2^ Robinson in 1998 in there study of 1000 patients come across only 2 open clavicle fracture.^4^ Same is true for Postachhini et al who observed 2 open fracture out of 535 patients in Italy in 2002.^5^

As true for open fracture, clavicle fracture also appear more common in younger population.^2^ It can be outside in or inside out type of open fracture similar to other fracture.^6^ Outside in type of fracture is usually observed after high energy trauma in acute setting. While inside out type of fracture appear either immediately or after sudden time duration following the course of skin indentation and gradually piercing of skin. It is usually the medial fragment of the clavicle which by pull of sternocleidomastoid get displaced superiorly and interrupt the skin.^7,8,9^ Our patient had a high energy trauma due to sharp object and presented acutely with open fracture. High energy trauma patient also present with associated injury with thoracic and head injury covering most of the scene followed by subclavian vessel injury and brachial plexus injury which are nearby.^2^ As high as 50% of open fracture had thoracic injury with dominance of pneumothorax observed in study conducted by Taitsman.^10^ Luckily our patient fall under rare group with no associated injuries despite high energy trauma.

Management starts in emergency room itself as that for all other fracture. In conjuction with obtaining X-ray chest or clavicle AP, starting of prophylactic IV antibiotics as per protocol of respective parts of world preferably within an hour of incident and tetanus toxoid is a must. Following that surgical irrigation and debridement within 6-12 hour of incident is preferred. Whether to fix the fracture or not to and that to in index or secondary setting is not well explained.^2^ Majority of fracture, 70% is fixed with implant in study by Taitsman.^10^ Also both the patient was fixed by Robinson.^4^ Either to proceed with internal or external fixation depends on the soft tissue status and contamination. Significant contamination and severe soft tissue damage is an indication for external fixation while its absence favour for internal fixation.^2,3,7^ Use of reconstruction locking compression as an external fixation with vacuum assisted closure has been successfully carried out by Kenyon et al and Norachart et al.^3,7^ While other cases are opted for internal fixation with anatomical clavicle locking plate. We after initial IV antibotics, wound irrigation and tetanus toxoid application proceeded with X-Ray. Then debridement was performed and fracture fixation done after four days of visit with anatomical locking plate.

Post op protocol is similar to closed fracture after plate fixation. Risk of infection and non union is believed to be higher than for closed fracture although not proven otherwise. Considering loss to follow up nearly 25% patient might have infection and 21% might present with nonunion as per Kenyon et al.^3^ Post operative X-ray of our patient shows implant insitu with maintenance of length and no displacement of fragment. While fourteenth post operative follow-up showed “good” clinical outcome as per Constant-Murley Shoulder outcome Score of 59.^11^

## CONCLUSION

Although clavicle fracture are common, it’s open variant are rare and are usually associated with high energy trauma usually as a part of poly-trauma. It should be treated different from closed clavicle fractures as the rate of infection and nonunion are high in open clavicle fractures as compared to closed ones so emphasis should be given on thorough initial debridement of wound while protecting the soft tissues as much as possible followed by proper wound care and antibiotics coverage and definitve fixation should be halted till there are no signs of infection and the wound is healthy.
